# *H2AFX* might be a prognostic biomarker for hepatocellular carcinoma

**DOI:** 10.1002/cnr2.1684

**Published:** 2022-07-29

**Authors:** Hailiang Hu, Tao Zhong, Suwei Jiang

**Affiliations:** ^1^ Department of Blood Transfusion The First Affiliated Hospital of Anhui Medical University Anhui China; ^2^ Department of Biological and Environmental Engineering Hefei University Hefei Anhui P. R. China

**Keywords:** biomarker, H2AFX, hepatocellular carcinoma, prognosis, survival analysis, tumor immunity

## Abstract

**Background:**

*H2AFX* can play a central role in DNA repair, replication, transcription regulation, and chromosomal stability. However, there is little research to explore the expression of *H2AFX* in cancers. Moreover, the correlation between the expression of *H2AFX* and tumor immunity, which affects the prognosis of hepatocellular carcinoma (HCC), is not clear. This article aimed to observe the correlation between *H2AFX* and tumor tissue infiltration biomarkers in HCC and its prognostic potential in HCC.

**Method:**

Oncomine and TIMER database were used to assess the expression level of *H2AFX* mRNA, and GEPIA and Kaplan–Meier databases were used to evaluate its prognostic potential. The TIMER database analyzed the relationship between h2afx expression level and tumor immune cell infiltration markers in liver cancer tissues.

**Results:**

The results showed that *H2AFX* was overexpressed in tumor tissues than normal tissues in HCC via analysis, and its expression level was correlated with the survival rate of HCC. Moreover, the expression level of *H2AFX* was related to various immune biomarkers. These results show that overexpression of *H2AFX* would reflect the poor prognosis of HCC, and these would also reflect that the gene *H2AFX* can affect the infiltration of HCC immune cells and then play a role in regulating tumor immunity.

**Conclusion:**

Our study showed that the gene *H2AFX* might be a potential poor prognostic biomarker in HCC and might be involved in the infiltration of HCC immune cells.

## 1 INTRODUCTION

Hepatocellular carcinoma (HCC) is 75% of the global primary liver cancer, one of the common subtypes of liver cancer.^1^ The incidence rate and mortality of liver cancer have recently decreased because of the improvement of early diagnosis and surgical techniques. However, the recurrence rate of liver cancer 2 years after an operation is 61.6%,^2^ and the metastasis rate of liver cancer is still very high.^3^ Thus, it is crucial to be aware of the molecular mechanism of the occurrence, development, and invasion of hepatocellular carcinoma and develop new assessment methods to improve the long‐term survival rate of patients.

DNA damage response (DDR) is an essential cellular protective mechanism. The failure of DNA damage repair may lead to disastrous cellular consequences, such as the occurrence and development of cancer.^4^ Many DNA damage repair pathways may significantly impact the prognosis and treatment response of different types of cancer.^5^ *H2AFX* is a potential regulator of DNA repair and is very important for DDR.^6^, ^7^ In some previous studies^8^, ^9^ *H2AFX* may help detect the transformation and progression of early HCC. *H2AFX* has been identified in various researches, including breast cancer,^10^ gastric intestinal metaplasia,^11^ and prostate cancer.^12^ The tumorigenesis and metastasis of HCC are regulated by the tumor microenvironment (TME),^13^ and immune disorders have been studied in HCC.^14^ The gene expression level in tumor tissue can reflect the prognosis of HCC, and these gene expression levels are also reliably related to the characteristics of TME.^15^ Studies had shown that the *H2AFX* expression level in fast‐growing tumors is significantly higher than that in slow‐growing tumors, indicating that they are related to the rapid growth of tumors,^8^ and the macrophage infiltration increased when the DSBs increased, including the expression of *H2AFX*.^16^ The previous study indicates a significant correlation between Macrophages M0 and *H2AFX*.^17^ *H2AFX* is a gene associated with B‐cell tumorigenesis,^18^ and Nbs1/γ‐H2AX/Brca1/Rad51 nuclear foci can be observed in activated B cells.^19^

This study aimed to investigate the relationship between *H2AFX* and tumor tissue infiltration biomarkers in HCC. Our study suggests the poor prognosis of patients with high expression of *H2AFX*, and the results showed that the *H2AFX* might play a positive role in the prognosis and the infiltration of tumor immune cells in HCC patients.

## 2 MATERIALS AND METHODS

### 2.1 Analyzed by oncomine

Oncomine^20^ (https://www.oncomine.org/resource/login.html) is a free platform to assess various gene expressions in various cancer, including 715 datasets and 86 733 samples. Oncomine can be used to excavate various new biomarkers in future research. In this manuscript, the gene *H2AFX* was the object gene, and its expression levels were compared between normal and cancer tissues. The threshold value of gene rank is set as the “top 10%,” and others are set as the “default.” The expression levels were significantly different between cancer and normal tissues when folding change >2, with a p‐value <.0001.

### 2.2 TNMplot analysis

TNMplot^21^ (https://tnmplot.com/analysis/) includes 56 938 unique multilevel quality‐controlled samples. The sources extracted from the databases include GEO, GTEx, TCGA, and TARGET databases. This manuscript uses RNA‐Seq data to analyze and include paired tumor and adjacent normal tissues and compare the *H2AFX* gene expression between tumor and normal tissue.

### 2.3 Kaplan–Meier plotter database analysis

Kaplan–Meier^22^ can recognize 70 632 gene symbols and assess the effect of 54 000 genes (including mRNA, miRNA, and protein) on survival in 21 types of cancer tissues. The sources extracted from the database include GEO, TCGA, and EGA databases. In this article, the *H2AFX* was inputted for searching according to the different clinical characteristics and analyzed all the samples with the following parameters: group cutoff is set “Median,” hazards ratio is set “yes,” 95% confidence interval is set “yes.”

### 2.4 Analyzed by GEPIA

GEPIA^23^ (http://gepia.cancer-pku.cn/) is a free online web used to analyze the RNA sequencing expression, including 9736 tumor tissues and 8587 normal sample tissues from the GTEx projects and TCGA database. GEPIA was used to analyze the overall and disease‐free survival curves via the log‐rank test and the Cox PH model. The group cutoff is median (cutoff‐high is set “50%,” cutoff‐low is set “50%”), add the 95% CI is set “dotted line,” the axis units are set “months.”

### 2.5 Analyzed by TIMER

The TIMER version 2.0 database^24^ (http://timer.comp-genomics.org/) analyzed immune infiltrates from diverse cancer types. The infiltration levels of immune cells were analyzed by TIMER, which is extracted from many TCGA samples (more than 40 tumor types). The *H2AFX* was inputted in the Gene_DE module for searching the different expression levels in tumor or normal tissues with default parameters. The gene *H2AFX* was inputted into the Gene module and then obtained the relationship between immune cell infiltration level and *H2AFX* expression in diverse tumors. Here, the gene *H2AFX* and immune‐associated cells were input, and then obtained the results, such as B cells, CD8 + T cells, CD4 + T cells, neutrophils, macrophages, and dendritic cells. Furthermore, the expression correlation between *H2AFX* and the biomarker genes of tumor‐infiltrating cells was analyzed, which gene expression values were transformed to log2 RSEM values.

## 3 RESULTS

### 3.1 The expression levels of *H2AFX* in diverse tumors

To understand the effect of expression of H2AFX and the *H2AFX* mRNA transcription levels were analyzed in various tumors and normal tissues via Oncomine.

**FIGURE 1:**
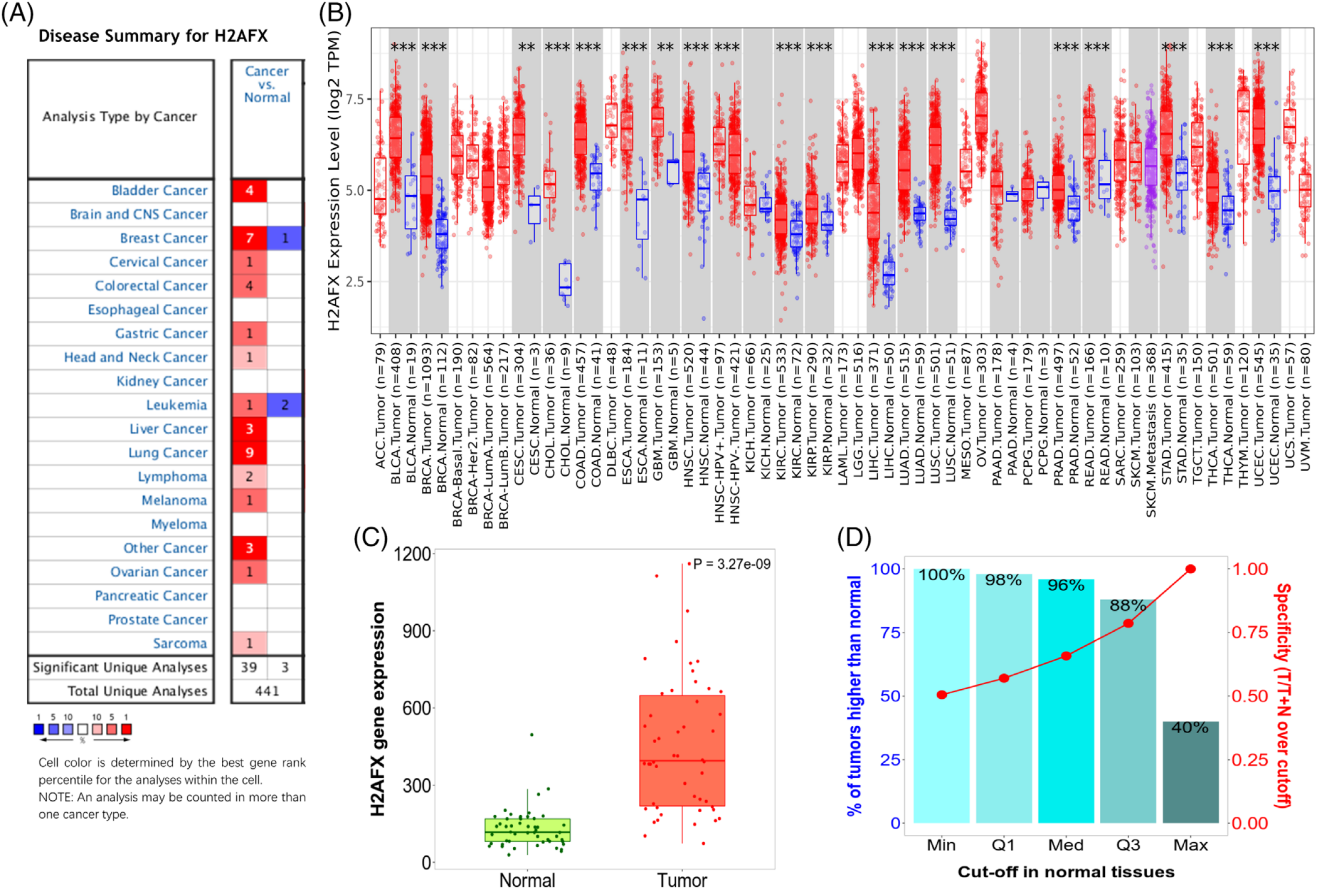
H2AFX expression levels in various types of cancer and normal tissues. (A) Expression levels of H2AFX in various cancer tissues or normal tissues via Oncomine analysis. (B) Expression levels of H2AFX in various cancer tumors via TIMER analysis. (C) and (D) H2AFX expression levels in liver hepatocellular cancer of cancer and normal tissues via TNMplot analysis.

Except for prostate cancer, pancreatic cancer, myeloma, kidney cancer, esophageal cancer, and brain cancer, the *H2AFX* expression was significantly upregulated in the tumor's tissues compared to the normal tissues, including liver, cervical, bladder, colorectal, breast, gastric, head and neck, leukemia, lung, lymphoma, melanoma, and sarcoma (Figure 1A). RNA‐seq data indicated that the *H2AFX* overexpression in 19 types of cancers via TIMER 2.0, including liver, bladder, breast, cervical, bile duct, colon, esophageal, glioblastoma multiforme, head and neck, kidney, lung, pancreatic, pheochromocytoma and paraganglioma, prostate, rectum, skin, stomach, thyroid, and uterine corpus (Figure 1B). The *H2AFX* gene expression levels are higher in tumors than in normal tissues (Figure 1C,D). Based on data presented in Figure 1, these data apple that H2AFX might play a positive role in developing or poor prognostic of HCC.

### 3.2 Effects of the over‐expression of *H2AFX* on the prognosis of patients are poor in HCC

The gene *H2AFX* expression levels were compared in the tumor and normal tissues, and the Kaplan Meier plotter evaluated the effect of its expression level on the prognosis of HCC. By comparing the overall survival (OS) rate, relapse survival (RS), poor post‐progression survival (PPS), and first progression survival (FPS). We found that *H2AFX* was overexpressed in various tumor tissues, leading to poor prognoses for different tumors, such as liver, breast, lung, and ovarian cancer. (Figure S1). *H2AFX* was overexpressed in different tumors and was analyzed via the GEPIA database. Moreover, overexpression of *H2AFX* was associated with low overall survival in various tumors (Figure S2).

**FIGURE 2:**
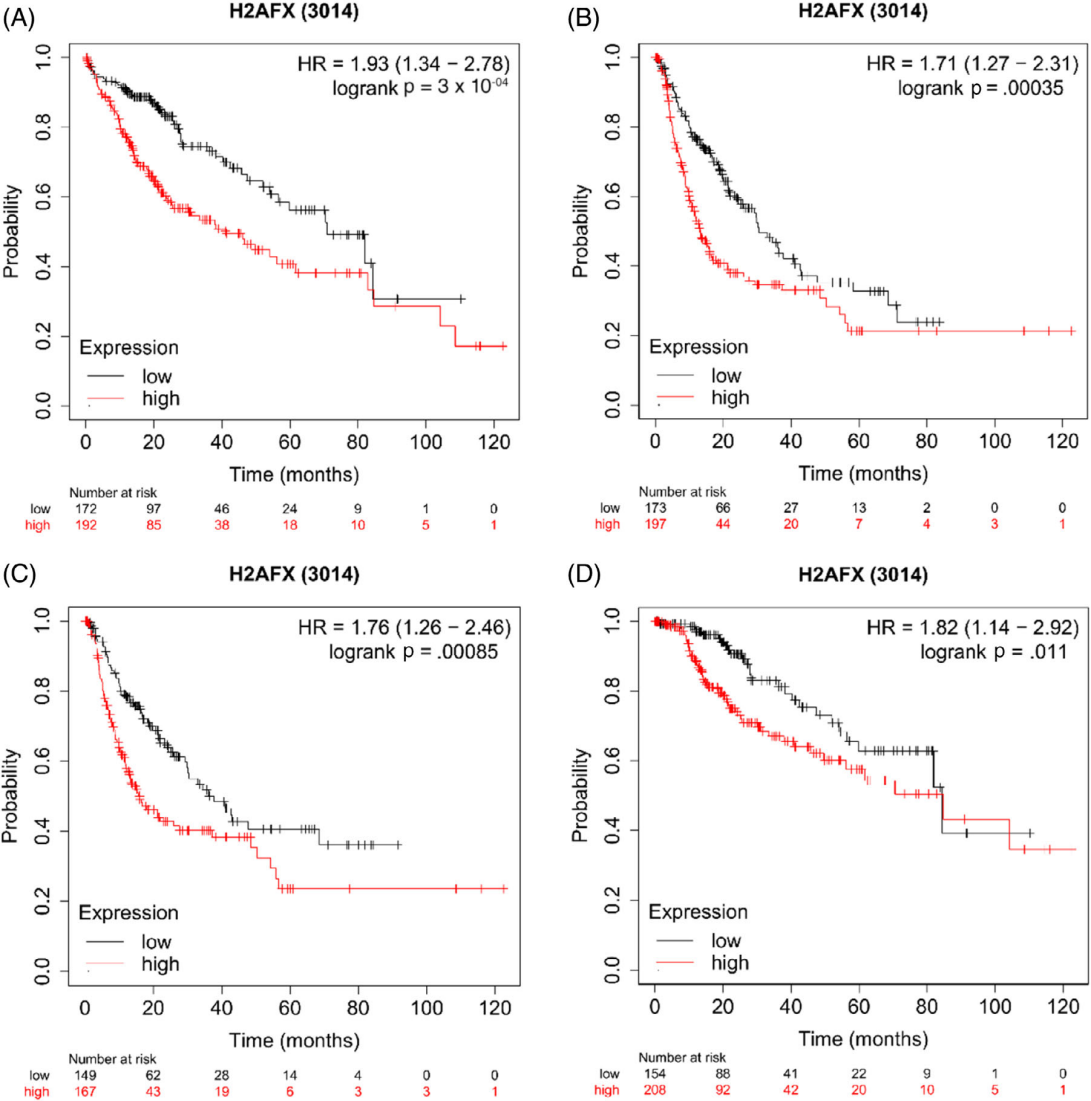
Effects of the over‐expression of H2AFX on prognostic survival in HCC patients via Kaplan–Meier plotter analysis. (A) Overall survival, (n = 364); (B) Progression free survival, (n = 370); (C) Relapse free survival, (n = 316); (D) Disease‐specific survival, (n = 362).

In generally, the results like the overall survival (Figure 2A, HR = 1.93[1.34–2.78], p = 3 × 10^‑04^), Progression free survival (Figure 2B, HR = 1.71[1.27–2.31], p = 3.5 × 10^‑04^), Relapse free survival (Figure 2C, HR = 1.76[1.26–2.46], p = 8.5 × 10^‑04^), Disease‐specific survival, (Figure 2D, HR = 1.82[1.14–2.92], p = 0.011) showed that were significantly decreased when the expression levels *of* gene *H2AFX* was over‐expression.

Furthermore, the correlation was analyzed between the overall survival and different clinical features by the Kaplan–Meier plotter database. (Table 1). The overall survival rate and progression‐free survival rate were compared from the following aspects: tumor stage or grade, alcohol consumption, hepatitis virus, gender, and Asian or another status, and the results suggest that overexpression of *H2AFX* may be one of the causes of poor progression.

**TABLE 1:** Effects of different pathological factors on the expression of *H2AFX* via Kaplan–Meier plotter.

Clinicopathological factors		OS (n = 364)				PFS (n = 370)			
		N	HR	p‐value	FDR	N	HR	p‐value	FDR
Stage	1	170	2.01 (1.07–3.77)	**.0262**	>50%	170	1.59 (0.96–2.62)	.067	100%
	2	83	2.35 (1.05–5.28)	**.033**	>50%	83	1.52 (0.72–3.18)	.27	100%
	3	83	2.5 (1.34–4.68)	**.003**	10%	83	2.18 (1.17–4.05)	**.012**	50%
	4	4				5			
Grade	1	55	3.57 (1.02–12.47)	**.034**	>50%	55	3.09 (1.14–8.35)	**.02**	50%
	2	174	1.77 (1.04–3.02)	**.032**	>50%	175	2.14 (1.38–3.31)	**.0005**	5%
	3	118	2.68 (1.19–6.02)	**.013**	>50%	119	1.47 (0.82–2.63)	.1951	100%
	4	12				12			
AJCC_T	1	180	2.1 (1.15–3.84)	**.014**	>50%	180	1.67 (1.03–2.7)	**.0366**	>50%
	2	90	2.07 (0.99–4.36)	**.049**	>50%	92	1.74 (0.86–3.49)	.1172	100%
	3	78	2.04 (1.1–3.77)	**.021**	>50%	78	2 (1.03–3.88)	**.037**	>50%
	4	13				13			
Vascular invasion	None	203	1.8 (1–3.25)	**.048**	>50%	204	1.54 (0.99–2.4)	.0545	100%
	Mirco	90	1.76 (0.81–3.81)	.15	100%	91	1.51 (0.83–2.76)	.177	100%
	Marco	16				16			
Gender	male	246	2.53 (1.48–4.33)	**.0005**	5%	246	1.84 (1.28–2.64)	**.0008**	10%
	female	118	1.56 (0.89–2.72)	.12	100%	120	1.57 (0.93–2.66)	.0908	100%
Race	White	181	1.48 (0.92–2.38)	.1	100%	183	1.68 (1.12–2.52)	**.011**	>50%
	Asian	155	11.57 (3.58–37.41)	**2.30 ×** ^**—07**^	1%	155	2.88 (1.68–4.94)	**6.10 × 10** ^**‑05**^	1%
Alcohol consumption	Yes	115	2.12 (1–4.49)	**.044**	>50%	115	2.07 (1.23–3.51)	**.0055**	20%
	None	202	2.13 (1.29–3.51)	**.0025**	20%	204	1.89 (1.24–2.86)	**.0023**	20%
Hepatits Virus	Yes	150	2.39 (1.05–5.45)	**.032**	>50%	152	1.6 (1–2.54)	**.046**	>50%
	None	167	2.18 (1.38–3.45)	**.0006**	5%	167	2.38 (1.5–3.77)	**.00015**	1%

*Note*: Bold values represent p < .05.

Abbreviations: HR, hazard ratio; OS, overall survival; PFS, progression‐free survival.

### 3.3 Effects of the over‐expression of *H2AFX* on immune cell infiltration level in HCC

**FIGURE 3:**
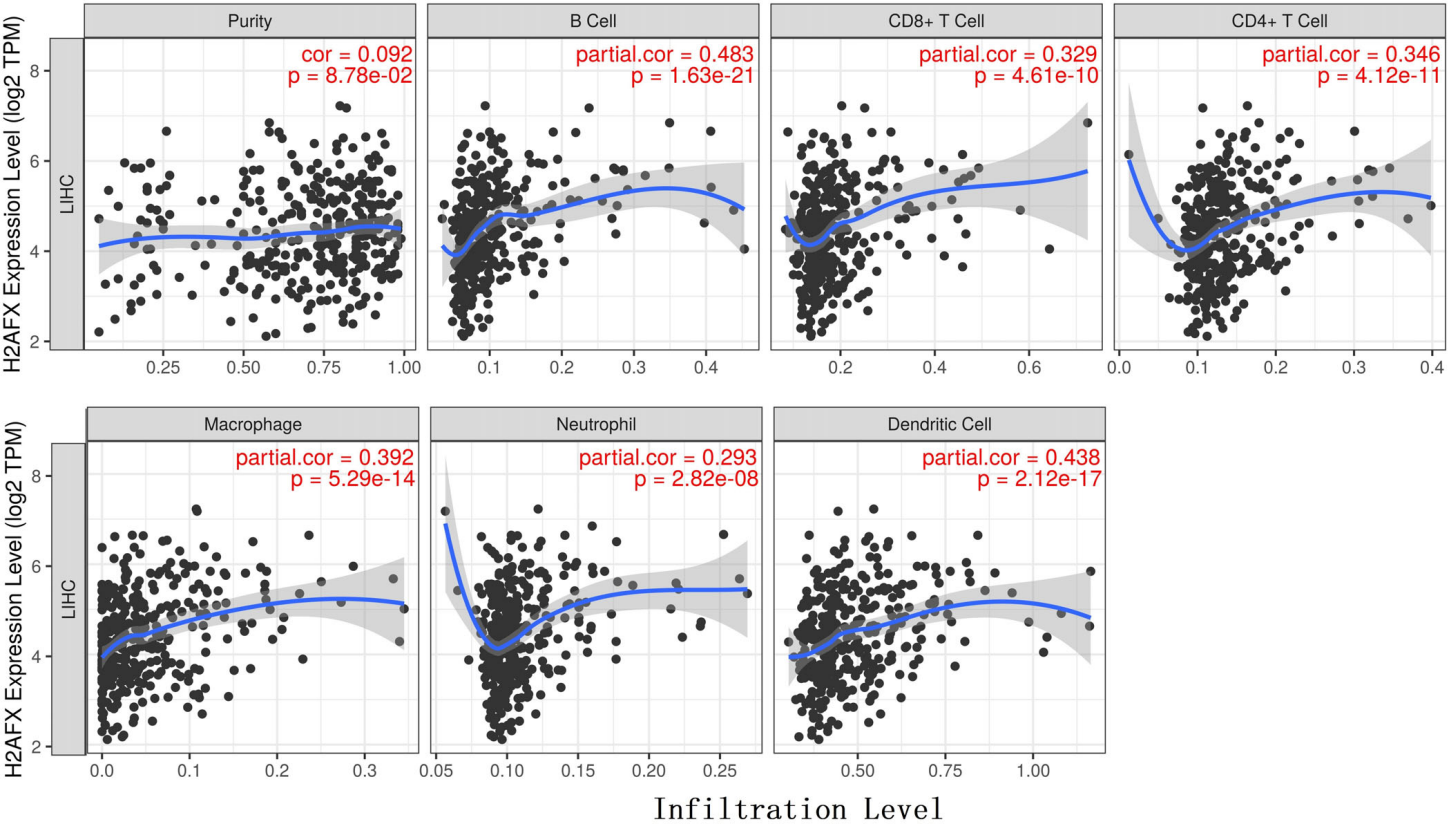
Effects of the over‐expression of H2AFX on immune cell infiltration levels via TIMER 2.0 analysis (n = 371).

We assessed the correlation between the expression level of *H2AFX* and the degree of immune cell infiltration level via TIMER 2.0 in 31 tumor tissues. And then, we observed a positive correlation with *H2AFX* over‐expression for some immune cells in various tumors (Table S1). *H2AFX* expression was positively correlation with B cells (Rho = 0.455, p = 4.77 × 10^‑19^), macrophage (Rho = 0.282, p = 1.02 × 10^‑07^), neutrophil (Rho = 0.162, p = 2.57 × 10^‑03^) and dendritic cell (Rho = 0.532, p = 1.26 × 10^‑26^) (Figure 3).

### 3.4 Effects of the over‐expression of *H2AFX* on biomarkers of various immune cell subsets

Finally, we selected some biomarkers in immune cells and analyzed their correlation with the expression level of H2AFX. The results showed that these biomarkers were positively correlated with the expression level of *H2AFX* in their subset of B cells, macrophage, neutrophil, and dendritic cell. We found that the gene *H2AFX* expression level was positively correlated with biomarkers via TIMER 2.0 analysis (such as CD19, CD22, CD79A, CCR2, PDGFB, CD80, IL1A, IL1B, ARG1, CD14, CD24, CXCR4, CD1A, CD83, HLA‐DPB1, HLA‐DQB1, ITGAX, and NRP1) (Table 2).

**TABLE 2:** Effects of the over‐expression of *H2AFX* on related genes and gene markers of immune cells.

Cell	Biomarkers	Purity	
		Correlation	p value
B cell	CD19	0.269	**1.45 × 10** ^**‑07**^
	CD22	0.181	**4.48 × 10** ^**‑04**^
	CD79A	0.232	**6.36 × 10** ^**‑06**^
	CD9	0.026	0.615
Tumor‐associated macrophage	CCL2	0.146	**6.47 × 10** ^**‑03**^
	CCR2	0.259	**1.03 × 10** ^**‑06**^
	CD40	0.101	6.18 × 10^‑02^
	PDGFB	0.218	**4.50 × 10** ^**‑05**^
M1 macrophage	CD80	0.330	**7.18 × 10** ^**‑11**^
	IL1A	0.345	**7.77 × 10** ^**‑12**^
	IL1B	0.314	**5.89 × 10** ^**‑10**^
	IL6	−0.011	8.36 × 10^‑01^
	NOS2	−0.131	**1.16 × 10** ^**‑02**^
	TLR2	0.165	**1.47 × 10** ^**‑03**^
	TLR4	−0.005	9.28 × 10^‑01^
M2 macrophage	ARG1	−0.388	**8.47 × 10** ^**‑15**^
	CD163	0.014	7.86 × 10^‑01^
	MS4A4A	0.035	5.02 × 10^‑01^
	PPARG	0.350	**3.87 × 10** ^**‑12**^
	VSIG4	0.057	2.73 × 10^‑01^
Neutrophils	CD14	−0.438	**8.47 × 10** ^**‑19**^
	CD24	0.402	**7.78 × 10** ^**‑16**^
	CXCR2	0.064	2.16 × 10^‑01^
	CXCR4	0.329	**8.30 × 10** ^**‑11**^
Dendritic cell	CD1A	0.390	**5.82 × 10** ^**‑15**^
	CD1C	0.152	**3.38 × 10** ^**‑03**^
	CD83	0.399	**1.34 × 10** ^**‑15**^
	HLA‐DPA1	0.145	**5.04 × 10** ^**‑03**^
	HLA‐DPB1	0.221	**1.81 × 10** ^**‑05**^
	HLA‐DQB1	0.209	**5.02 × 10** ^**‑05**^
	HLA‐DRA	0.178	**5.66 × 10** ^**‑04**^
	ITGAX	0.334	**3.96 × 10** ^**‑11**^
	NRP1	0.224	**1.36 × 10** ^**‑05**^

*Note*: Bold values indicate p < .05.

## 4 DISCUSSION

Hepatocellular carcinoma (HCC) is a highly vascular tumor, is the sixth most prevalent cancer and the second fatal tumor globally, and its incidence rate continues to rise.^25^ Only a few patients with HCC sensitive to radiotherapy and chemotherapy can be treated in time due to early diagnosis; however, the prognosis is poor due to missing the best treatment time.^26^ The public resource databases were used in this study to analyze the relationship between *H2AFX* and HCC via the bioinformatics analysis method. Our results showed that *H2AFX* was overexpressed in HCC and was positively correlated with the poor survival rate of HCC patients. In addition, the gene *H2AFX* overexpression in HCC patients was positively correlated with various immune biomarkers and immune cell infiltration. These results suggest that *H2AFX* may be involved in the regulation of tumor immunity by regulating the immune cells infiltration in HCC.

In some studies, the mRNA was used to analyze the prognosis because the mRNA with different expression levels in different tissues can be used as early cancer monitoring markers or prognostic monitoring markers. The mRNA expression levels of *H2AFX* were analyzed in normal, and tumor tissues showed significantly upregulated in various tumors, and the expression pattern was similar in HCC among different databases. H2AX is a variant of histone H2A, and its expression levels significantly differ in various cell lines and tumor tissues.^27^ A previous study^28^ applied the regulation of H2AX, and its interacting proteins may play a role in tumor immune interaction and inducing tumor phenotype (including proliferation and migration) and play a synergistic role in tumorigenesis.^29^ It is reported that mice with H2AX gene knockout have increased genomic instability and higher cancer risk.^30^ Some evidence suggests that the regulatory level of γ‐H2AX may be tissue‐specific.^31^, ^32^ Some research^33^, ^34^ suggested that the *H2AFX* variant is associated with an increased risk of gastric and breast cancer. Although the role of *H2AFX* in DDR is determined, its role in tumorigenesis is still unclear. A study^17^ shows that *H2AFX* was involved in immune infiltration and metastasis. Over‐expression of *H2AFX* was associated with poor prognosis in HCC. Therefore, evaluating the expression level of *H2AFX* has a particular suggestive effect on tumor prognosis.

Four important survival parameters were taken as reference objects, and the gene *H2AFX* expression level was analyzed on 371 patients' prognosis in HCC by Kaplan–Meier plotter. Here, we found that the *H2AFX* over‐expression levels were correlated with the low overall survival and disease‐specific survival of patients with liver cancer, suggesting that over‐expression of *H2AFX* mRNA will reduce the HCC patients survival with liver cancer and may reduce the resistance of patients to other diseases. Besides, the results of PFS showed that high expression of *H2AFX* could enhance the further deterioration of HCC. We also found the results of relapse‐free survival. We found a similar trend in relapse‐free survival, suggesting that the over‐expression of *H2AFX* is still negatively correlated with the prognosis of HCC patients treated with various treatment methods. Therefore, these results reflect that *H2AFX* may be a potential biomarker of poor prognosis in HCC. The trend of relapse‐free survival has a similar conclusion, indicating that the overexpression of *H2AFX* could reflect the negative correlation between the prognosis of HCC patients after various treatment methods. So *H2AFX* may become a potential poor prognostic biomarker in HCC.

There are abundant macrophages in the TME, promoting tumor invasion, metastasis, and angiogenesis and increasing immunosuppression.^35^ There is extensive immune cell infiltration in HCC patients.^36^ The poor prognosis of HCC is often related to the high content of B cells, CD8 + T cells, dendritic cells, neutrophils, and macrophages.^37^ The expression of *H2AFX* is highly correlated with the expression of immune cell markers, such as markers of B cells, Tam, neutrophils, and NK cells, suggesting that *H2AFX* has a potential function in regulating the infiltration and activity of immune cells. B cell infiltration can enhance angiogenesis and poor prognosis.^38^ Neutrophil infiltration can be one of the driving factors for the poor prognosis of HCC.^39^ Similarly, dendritic cells can activate T lymphocytes and play an essential role in regulating adaptive immune response and antitumor effects^40^, ^41^, ^42^ The over‐expression of TAMs is closely associated with a low prognosis survival rate^43^ and promotes tumor metastasis through various mechanisms.^44^ The high activity of TAM infiltration is consistent with the overexpression of *H2AFX* in HCC, indicating that *H2AFX* can also affect the occurrence and progression of HCC by increasing the overexpression of TAM. In addition, our finding is that the expression of *H2AFX* is related to the expression of macrophage (M1 and M2) markers, indicating its potential impact on tumor prognosis in terms of macrophage secretion of cytokines. The macrophage can encourage tumor cells to escape into the circulatory system, inhibit antitumor immune mechanism and response, and promote adaptive immune response.^45^ The various subtypes of macrophages play different roles in TME. Such as M1 may produce high levels of pro‐inflammatory cytokines, which promote inflammation, sterilization, and antitumor, while M2 promotes tumor progression and contributes to tumor angiogenesis, growth, and metastasis.^46^ The previous study inferred that H2AFX might play an important role in tumor metastasis.^17^ In this study, some biomarkers' expression level was negatively correlated with the overexpression of *H2AFX* in macrophages. This inconsistency may be caused by the complexity of the tumorigenesis and immune mechanisms. These results reflect the complexity of tumor pathogenesis. It further applied that *H2AFX* may be involved in the occurrence and progression, which proves that *H2AFX* is a potential biomarker gene with poor prognosis of HCC.

The results showed that the association between *H2AFX* and IL1 and CD80 (biomarkers of M1 macrophages) was very high, suggesting that IL1 and CD80 may also be potential target genes for treating HCC. In addition, the overexpression of *H2AFX* is also highly correlated with some biomarker genes in neutrophils and dendritic cells (such as CD14, CD24, CD1a, and CD1c), which may also become a new research target in the future. The data were obtained in various databases and analyzed through various methods. However, our results can only provide some preliminary theoretical basis, and accurate, empirical evidence must be obtained through follow‐up experiments and clinical verification.

## AUTHOR CONTRIBUTIONS

**hailiang hu:** Conceptualization (lead); writing – original draft (lead); writing – review and editing (supporting). **tao zhong:** Formal analysis (equal); software (equal). **suwei jiang:** Conceptualization (lead); formal analysis (lead); supervision (lead); writing – review and editing (lead).

## CONFLICT OF INTEREST

The authors report no conflict of interest.

## Supporting information


**Figure S1** Effects of the over‐expression of H2AFX on prognostic survival in four types of cancers via Kaplan–Meier plotter analysis. (A–C) The survival curve of overall survival, relapse free survival and post progression survival in breast cancer (n = 1879, n = 4929, n = 458). (D–F) The survival curve of overall survival, first progression survival and post progression survival in gastric cancer (n = 875,n = 640, n = 498). (G‐I) The survival curve of overall survival, first progression survival and post progression survival in lung cancer (n = 1925, n = 982, n = 344). (J‐L) The survival curve of overall survival, progression free survival and post progression survival in the ovarian cancer (n = 1656, n = 1435, n = 782).

**Figure S2** Effects of the over‐expression of H2AFX on prognostic survival in different types of cancers via GEPIA analysis. (A‐B) The survival curve of overall survival and Disease free survival in liver hepatocellular carcinoma (LIHC) (A‐B), brain lower grade glioma (LGG) (C‐D), kidney renal clear cell carcinoma (KIRC) (E‐F), kidney renal papillary cell carcinoma (KIRP) (G‐H), adrenocortical carcinoma (ACC) (I‐J), lung adenocarcinoma (LUAD) (K‐L), Mesothelioma (MESO) (M‐N), pheochromocytoma and paraganglioma (PCPG) (O‐P), prostate adenocarcinoma (PRAD) (Q‐R), sarcoma (SARC) (S‐T), testicular germ cell tumors (TGCT) (U‐V), uveal melanoma (UVM) (W‐X)

**Table S1** Effects of different immune cell infiltration levels on the expression of H2AFX via TIMER 2.0 analysis

## Data Availability

Data sharing does not apply to this article as no new data were created or analyzed in this study. The data supporting this study are openly available in a different database.
