# Supplementary material for: H2AFX might be a prognostic biomarker for hepatocellular carcinoma
Source: Cancer Rep (Hoboken). 2022 Jul 29;6(1):e1684. doi: 10.1002/cnr2.1684 (PMC9875689; doi:10.1002/cnr2.1684)
Supplement: Supplementary file 1 — Figure S1 Effects of the over‐expression of H2AFX on prognostic survival in four types of cancers via Kaplan–Meier plotter analysis. (A–C) The survival curve of overall survival, relapse free survival and post progression survival in breast cancer (n = 1879, n = 4929, n = 458). (D–F) The survival curve of overall survival, first progression survival and post progression survival in gastric cancer (n = 875,n = 640, n = 498). (G‐I) The survival curve of overall survival, first progression survival and post progression survival in lung cancer (n = 1925, n = 982, n = 344). (J‐L) The survival curve of overall survival, progression free survival and post progression survival in the ovarian cancer (n = 1656, n = 1435, n = 782). [file CNR2-6-e1684-s003.docx]

**Supplement figure 1**


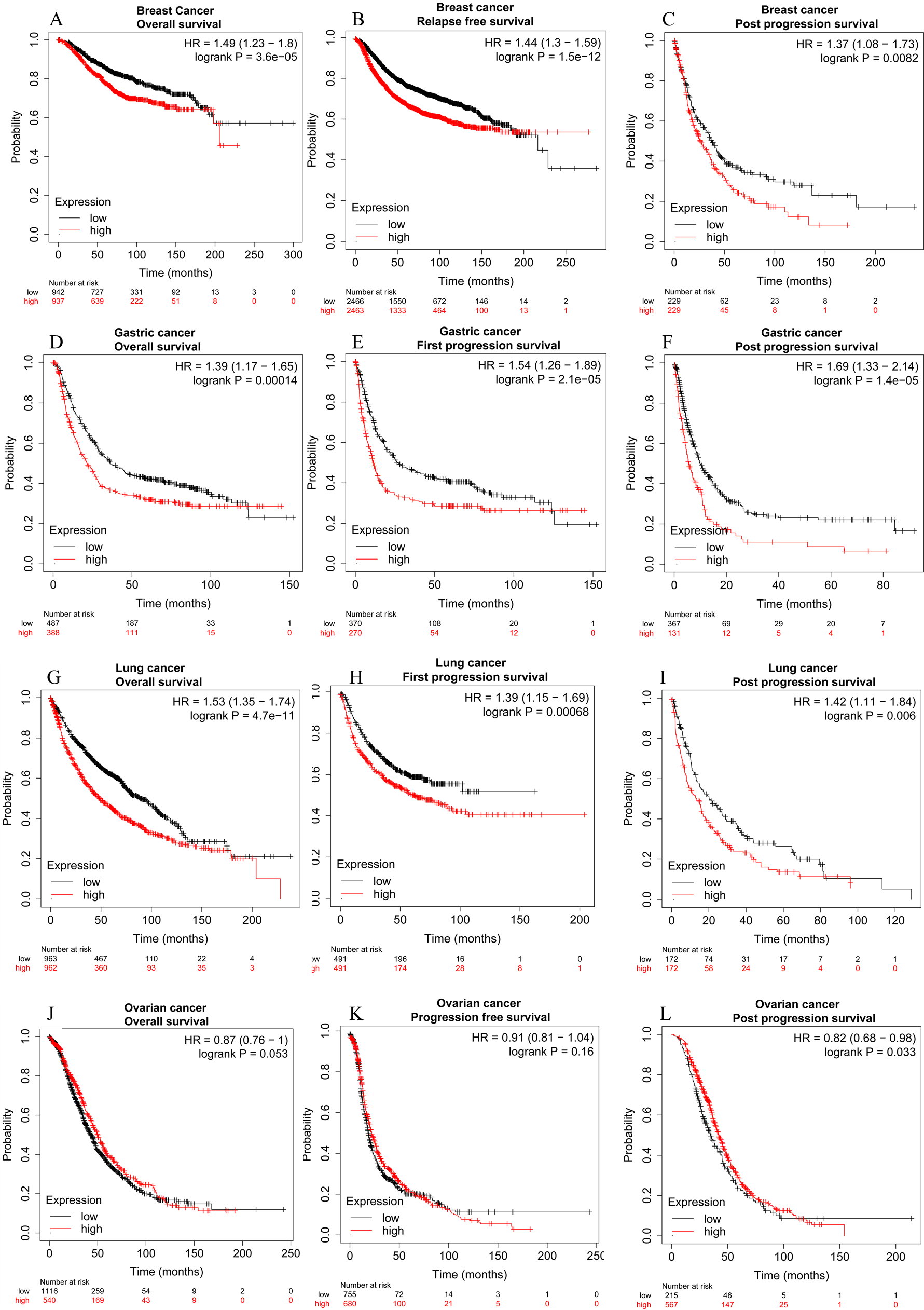


Supplement figure 1. Effects of the over-expression of H2AFX on prognostic survival in four types of cancers via Kaplan-Meier plotter analysis. (A–C) The survival curve of overall survival, relapse free survival and post progression survival in breast cancer (n =1879, n =4929, n=458). (D–F) The survival curve of overall survival, first progression survival and post progression survival in gastric cancer (n =875,n=640, n =498). (G-I) The survival curve of overall survival, first progression survival and post progression survival in lung cancer (n =1925, n=982, n =344). (J-L) The survival curve of overall survival, progression free survival and post progression survival in the ovarian cancer (n=1656, n=1435, n=782).
