# Supplementary material for: H2AFX might be a prognostic biomarker for hepatocellular carcinoma
Source: Cancer Rep (Hoboken). 2022 Jul 29;6(1):e1684. doi: 10.1002/cnr2.1684 (PMC9875689; doi:10.1002/cnr2.1684)
Supplement: Supplementary file 2 — Figure S2 Effects of the over‐expression of H2AFX on prognostic survival in different types of cancers via GEPIA analysis. (A‐B) The survival curve of overall survival and Disease free survival in liver hepatocellular carcinoma (LIHC) (A‐B), brain lower grade glioma (LGG) (C‐D), kidney renal clear cell carcinoma (KIRC) (E‐F), kidney renal papillary cell carcinoma (KIRP) (G‐H), adrenocortical carcinoma (ACC) (I‐J), lung adenocarcinoma (LUAD) (K‐L), Mesothelioma (MESO) (M‐N), pheochromocytoma and paraganglioma (PCPG) (O‐P), prostate adenocarcinoma (PRAD) (Q‐R), sarcoma (SARC) (S‐T), testicular germ cell tumors (TGCT) (U‐V), uveal melanoma (UVM) (W‐X) [file CNR2-6-e1684-s001.docx]

**Supplement figure 2**


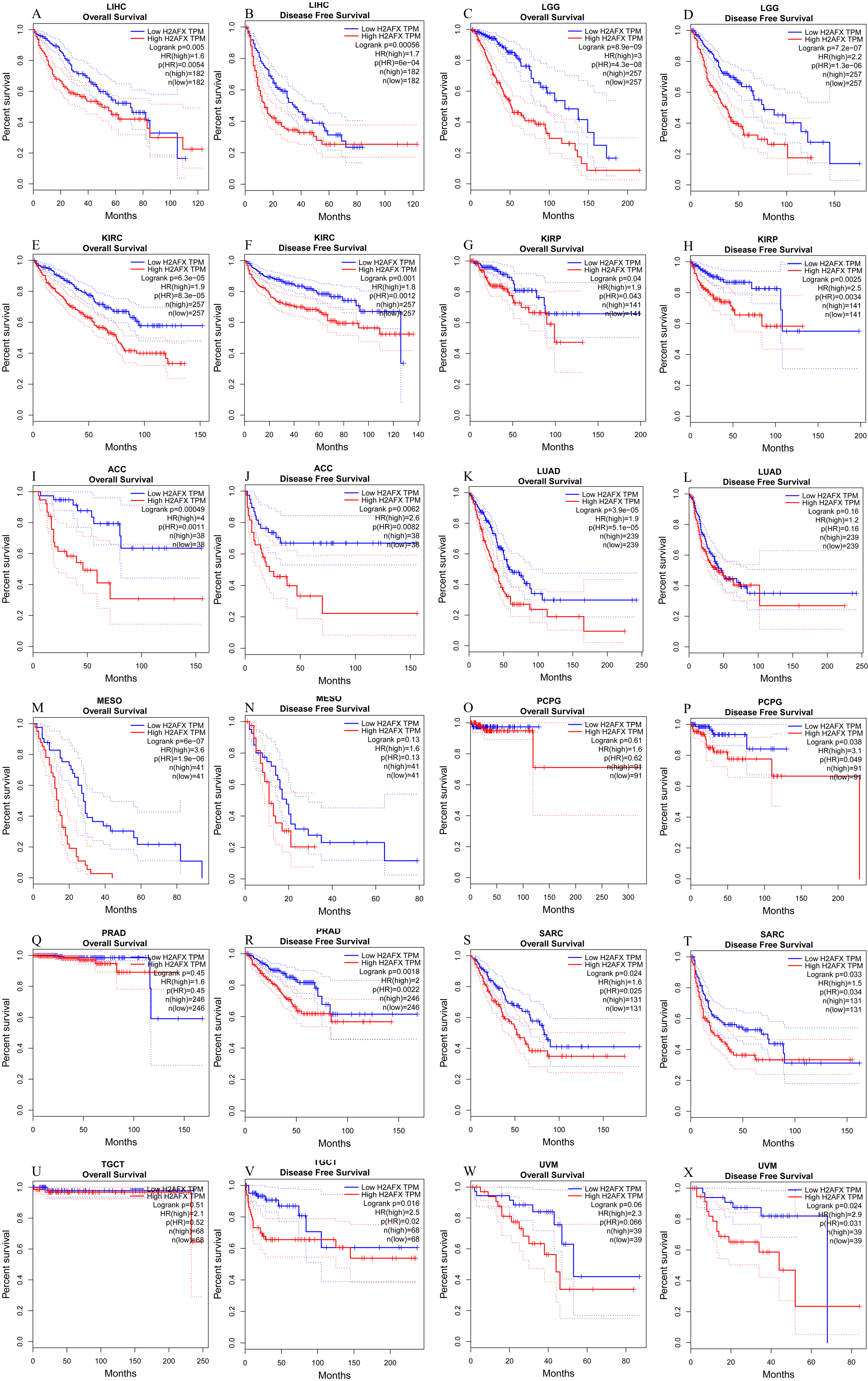


Supplement figure 2. Effects of the over-expression of H2AFX on prognostic survival in different types of cancers via GEPIA analysis. (A-B) The survival curve of overall survival and Disease free survival in liver hepatocellular carcinoma (LIHC) (A-B), brain lower grade glioma (LGG) (C-D), kidney renal clear cell carcinoma(KIRC) (E-F), kidney renal papillary cell carcinoma(KIRP)(G-H), adrenocortical carcinoma(ACC)(I-J), lung adenocarcinoma(LUAD)(K-L), Mesothelioma(MESO)(M-N), pheochromocytoma and paraganglioma(PCPG)(O-P), prostate adenocarcinoma(PRAD)(Q-R), sarcoma(SARC)(S-T), testicular germ cell tumors(TGCT)(U-V), uveal melanoma(UVM)(W-X)
