# Supplementary material for: H2AFX might be a prognostic biomarker for hepatocellular carcinoma
Source: Cancer Rep (Hoboken). 2022 Jul 29;6(1):e1684. doi: 10.1002/cnr2.1684 (PMC9875689; doi:10.1002/cnr2.1684)
Supplement: Supplementary file 3 — Table S1 Effects of different immune cell infiltration levels on the expression of H2AFX via TIMER 2.0 analysis [file CNR2-6-e1684-s002.docx]

| Supplement table 1 Effects of different immune cell infiltration levels on the expression of H2AFX via TIMER 2.0 analysis | | | | | | | |  |
| --- | --- | --- | --- | --- | --- | --- | --- | --- |
| cancer | Purity | B cell | T cell CD8+ | T cell CD4+ | Macrophage | Neutrophil | dendritic cell | |
| ACC (n=79) | **0.344** | 0.026 | 0.141 | 0.177 | 0.102 | **0.297** | -0.117 | |
| BLCA (n=408) | 0.090 | 0.083 | **0.115** | -0.060 | 0.029 | 0.017 | 0.075 | |
| BRCA (n=1100) | **0.123** | **0.176** | **-0.236** | **0.121** | **-0.238** | 0.019 | **0.093** | |
| CESC (n=306) | **0.160** | -0.002 | -0.062 | 0.073 | -0.008 | **-0.215** | **-0.122** | |
| CHOL (n=36) | -0.204 | -0.222 | -0.286 | -0.135 | -0.159 | **-0.349** | -0.025 | |
| COAD (n=458) | 0.067 | **0.284** | 0.037 | **-0.122** | **-0.261** | -0.030 | -0.074 | |
| DLBC (n=48) | 0.282 | -0.175 | 0.024 | **-0.385** | 0.073 | 0.056 | -0.275 | |
| ESCA (n=185) | **0.278** | 0.031 | **-0.153** | 0.096 | **-0.163** | -0.137 | 0.055 | |
| GBM (n=153) | **0.503** | 0.121 | **-0.210** | 0.081 | -0.112 | **-0.352** | **-0.209** | |
| HNSC (n=522) | **0.157** | **-0.209** | **-0.166** | **0.253** | **-0.167** | **0.136** | **-0.105** | |
| KICH (n=66) | -0.017 | -0.041 | -0.125 | **0.448** | 0.069 | 0.017 | **0.255** | |
| KIRC (n=533) | **-0.161** | -0.028 | **0.137** | 0.062 | **-0.188** | 0.081 | **0.251** | |
| KIRP (n=290) | -0.040 | **0.199** | 0.047 | **0.461** | **-0.162** | **0.207** | **0.137** | |
| LGG (n=516) | -0.020 | **0.167** | **-0.259** | **0.412** | **0.164** | **0.229** | **0.316** | |
| LIHC (n=371) | 0.092 | **0.455** | 0.069 | **0.321** | **0.282** | **0.162** | **0.532** | |
| LUAD (n=515) | 0.053 | **-0.113** | 0.007 | -0.014 | 0.004 | 0.077 | -0.039 | |
| LUSC (n=501) | **0.270** | 0.054 | **-0.112** | 0.016 | 0.026 | **-0.210** | **-0.155** | |
| MESO (n=87) | -0.039 | **0.226** | -0.019 | 0.198 | 0.121 | -0.120 | **0.241** | |
| OV (n=303) | **0.146** | -0.045 | -0.117 | **0.136** | -0.007 | 0.065 | 0.077 | |
| PAAD (n=179) | 0.104 | 0.117 | **-0.230** | **0.198** | **-0.327** | -0.095 | **-0.165** | |
| PCPG (n=181) | 0.147 | -0.030 | -0.052 | -0.048 | -0.134 | 0.112 | 0.022 | |
| PRAD (n=498) | **0.140** | -0.043 | **-0.188** | -0.026 | **-0.123** | 0.024 | -0.012 | |
| READ (n=166) | 0.139 | -0.068 | **-0.214** | -0.131 | -0.114 | -0.092 | -0.108 | |
| SARC (n=260) | 0.065 | **0.129** | 0.082 | -0.070 | -0.031 | 0.053 | 0.113 | |
| SKCM (n=471) | -0.023 | **0.096** | **-0.179** | **0.147** | -0.011 | -0.059 | **0.133** | |
| STAD (n=415) | **0.130** | 0.082 | -0.043 | -0.054 | **-0.329** | **-0.160** | 0.016 | |
| TGCT (n=150) | **0.295** | **-0.247** | **-0.264** | -0.105 | **-0.246** | **-0.200** | **-0.165** | |
| THCA (n=509) | -0.015 | -0.051 | **-0.096** | **0.108** | -0.050 | -0.037 | 0.017 | |
| THYM (n=120) | -0.182 | **0.603** | **0.865** | **0.641** | **-0.295** | **0.210** | **0.797** | |
| UCEC (n=545) | 0.078 | -0.031 | -0.168 | -0.075 | -0.003 | -0.109 | -0.132 | |
| UCS (n=57) | 0.073 | -0.115 | -0.238 | **0.293** | -0.023 | -0.182 | -0.195 | |
| UVM (n=80) | 0.117 | **-0.302** | 0.182 | **-0.232** | -0.192 | -0.145 | **-0.261** | |
| Red bold values represent positive correlation(P<0.05, P>0), green bold values represent negetive correlation(P<0.05, P>0) | | | | | | | |  |
